# Supplementary material for: Simplified antibiotic regimens for young infants with possible serious bacterial infection when the referral is not feasible in the Democratic Republic of the Congo
Source: PLoS One. 2022 Jun 30;17(6):e0268277. doi: 10.1371/journal.pone.0268277 (PMC9246187; doi:10.1371/journal.pone.0268277)
Supplement: S1 Table — (PDF) [file pone.0268277.s001.pdf]

Supporting information

S1 Table. Dose and frequency of oral amoxicillin

	**Oral amoxicillin**<br>Given twice daily for 7 days
**Weight (kg)**	**Dispersible tablet**<br>(250 mg) per dose
**1.5 - 2.4**	**1/2**
**2.5 – 3.9**	**1/2**
**4.0 – 5.9**	**1**
