# Supplementary material for: Simplified antibiotic regimens for young infants with possible serious bacterial infection when the referral is not feasible in the Democratic Republic of the Congo
Source: PLoS One. 2022 Jun 30;17(6):e0268277. doi: 10.1371/journal.pone.0268277 (PMC9246187; doi:10.1371/journal.pone.0268277)
Supplement: S2 Table — (PDF) [file pone.0268277.s002.pdf]

Supporting information

S2 Table. Dose and frequency of injectable antibiotics

	**Gentamicin**<br>(Strength 20 mg/mL)<br>Given once daily	**Ampicillin**<br>(Strength 250 mg/1,5 ml)<br>Given twice daily on an outpatient basis
**Weight (kg)**	**Volume par dose (mL)**	**Volume par dose (mL)**
**1.5 - 2.4**	**0.4**	**0.8**
**2.5 – 3.9**	**0.8**	**1.2**
**4.0 – 5.9**	**1.2**	**1.5**
