# Supplementary material for: Simplified antibiotic regimens for young infants with possible serious bacterial infection when the referral is not feasible in the Democratic Republic of the Congo
Source: PLoS One. 2022 Jun 30;17(6):e0268277. doi: 10.1371/journal.pone.0268277 (PMC9246187; doi:10.1371/journal.pone.0268277)
Supplement: Attachment — Submitted filename: Response_to_editor_and_reviewers_November_04_2021_Final.docx [file pone.0268277.s004.docx]

Mehreen Arshad, M.D.

Academic Editor

PLOS ONE

Re: PONE-D-20-38722

Simplified antibiotic regimens for young infants with possible serious bacterial infection when referral is not feasible in the Democratic Republic of the Congo

Dear Dr Arshad:

We thank you and the reviewers for your review of our manuscript. Because of the positive nature of the review, and at your request, we are submitting a revised manuscript. The following is a detailed list of the revisions and/or our responses.

We believe that our responses to the helpful advice from the reviewers and subsequent edits have improved the manuscript substantially. We hope that you will now deem it acceptable for publication. We look forward to your further consideration.

Sincerely,

Adrien Lokangaka, on behalf of the authors

**Comments from Reviewer #1**

**The authors have highlighted an important topic considering the problem of limited health care access to underprivileged population in the region. The manuscript has written in detail. However, I am unable to grab information on the Qualitative component of the project.**

*We apologize for the confusion about the methodological components of the study. Below we respond to specific questions and have amended the text to add clarity.*

**Methods:
Comment 1: They have mentioned that it was the mixed method study but how the study was conducted whether qualitative component conducted first or the Quantitative component ? i.e. is it the sequential mixed method study design? please clarify this in the methodology
It seems that during implementation of intervention they have conducted FGDS and KII and table 4 were their results but how many FGDs and KII conducted? this information is missing**.

*We use the term “mixed method” because both qualitative and quantitative methods were used in the study. Qualitative methods, including focus group discussions and in-depth interviews, were used during the situational analyses and were integral in the development of the implementation strategy. In the original manuscript, the order of events and details of the focus group discussions were not included. These details have been added to the Methods section under a new heading, “Barriers to health care seeking”.* *The barriers discovered during these analyses are listed in a new table, Table 2. This information is included in the Methods section because it was collected during the preparatory phase and informed the methods. The main thrust of this publication is the evaluation of the strategy (i.e., primary and secondary outcomes), and this was entirely quantitative.*

**Results:

Comment 2: This component is too long, authors have described each and everything in the text. it can be shortened by mentioning only key information in the text and for other variables it is better to direct the reader to the table.**

*Thank you for this helpful suggestion. We have condensed the Results section by reducing the amount of detail in the text as suggested.*

**Comment 3: There is discrepancy in proportions in text and tables. Refer to table 2 on page 17 and text in lines 385 and 394 on page 18**

*The text now corresponding to line 370 has been revised slightly, and the discrepancy corrected: “Severe pneumonia was identified in 141 infants, who were all referred to a hospital, but 90.8% families refused referral advice and accepted treatment at the health centre (now Table 3).”*

*The text now corresponding to line 378 has been revised, and the discrepancy corrected: “Health centre nurses classified 52 infants as critical illness, referred them to a hospital, and 86.5% of families accepted referral advice (now Table 3).” The figure in Table 3 has been corrected.*
 **Tables:
Comment 4: if operational definitions of pneumonia, severe pneumonia and CSI or critical illness are mentioned in the methods then it can be removed from table 3 and 4 footnotes.**

*These repeated definitions have been removed from Tables as advised.*

**Comments from Reviewer #2**

**1- It may be important to precise the proportion of neonatal infections due to bacterial infections worldwide and/or in LMIC/DRC to appreciate the importance of the implementation of outpatient treatment for possible serious bacterial infections in young infants.**

*To our knowledge, precise information about the relative prevalence of bacterial proportion of community acquired neonatal sepsis worldwide, especially from the LMICs, is limited. Viral infections also cause neonatal sepsis. Most bacterial data is available from tertiary care hospitals, which does not provide a good idea about community prevalence. Recently, ANISA, a community-based study from South-East Asia, reported a cause in 26% of the PSBI episodes (16% bacterial and 12% viral) (Saha et al. Lancet 2018). Among babies who died, the cause was attributed to 46% of PSBI episodes, among which 92% were bacterial. Such data from Africa, including the DRC is unavailable.*

**2-The authors did not indicate the proportion of patients identified by CHW in the study area. This information could be useful to evaluate their importance as with the current data this might be underestimated.**

*It is difficult to say it precisely, but we believe that most infants identified and referred by CHWs were from the study area. However, we cannot exclude the possibility of some referrals by CHWs from outside the study catchment area, as seen by a large number of infants (n=426) who sought care from the health centres located in the study catchment area.*

**3- If we assume that the CHW work only in their health areas, all patients from outside the study area should be brought by their family to the health centers. So how could we have 426 patients from outside the study area and only 385 walk-in patients?**

*It is correct that CHWs in the study catchment area did not typically work outside of their coverage area that was limited to 5-7 km around the health centre. As reported in situation analysis in the manuscript, in routine settings, the health centres did not have a regular supply of essential medicines and faced stockouts. Many families from outside the study catchment area sought care for their sick young infants at health centres participating in the study when they became aware of free treatment and a regular supply of medicines through the research project. Similarly, CHWs working outside of the study area were also aware of this information and referred some of the sick infants to the health centres in the study area. We presume that some of these referrals were made through a local CHW.*

**4- A total of total of 285 young infants with PSBI were identified in the study area and 346 were identified by the CHW. Did the CHW and/or nurses from other health areas also referred patients to health centers in the study area? Adrien, this is a good question.**

*Please see the responses to the above two comments. Some families outside the catchment area went directly to the nearby health centre, and others went there through CHWs working outside the study area.*

**5- The authors have identified some challenges to the implementation of their protocol and have adopted certain solutions at the beginning of the study. However, there is no mention of the appropriateness and/or evaluation of these solutions: any delay in the transportation of patients due to unavailability of HCW or insufficient number of bikes (which might have influenced the outcome of patients treated at the district hospital)? Stock-outs of medications despite the expected higher number of patients?**

*We do not have granular data on specific barriers to implementation. We attempted to describe comprehensively the entire implementation strategy, including facilitators of implementation (e.g., training, job aids and commodities and financial subsidies for care). However, our data collection focused only on the results of the strategy (e.g., number of referrals and success of treatment). Therefore, we cannot comment upon the impact of specific actions.*

**6- The mortality in the group of participants treated in the referred hospital was higher compared to those treated at home or at the health centers. Moreover, this mortality is higher compared to previous studies. The authors have provided some interesting explanations to these findings; however, could the difference between the two groups also explain these results? For example, the distribution of comorbidities in the two groups, families with sicker patients more likely to accept referral.**

*The reviewer offers a very plausible and likely explanation. We have added text to the Discussion section to highlight this possibility.*

**7-Line 108: please define the acronym “PSBI” on its first occurrence in the text.**

*The definition has been added in the first paragraph of the introduction section, where PSBI is mentioned for the first time.*

**Comments from Reviewer #3 (We assume that the comments from this Reviewer appear in the pdf attached to email from the editor.)**

**The manuscript is easy to understand, very descriptive, informative and generally well-written. Minor changes mainly relating to grammatical errors are required. The introduction should also be expanded to provide comprehensive information on other factors that may induce serious bacterial infections in infants such as the mother`s immunity and neonatal immunisation.**

*Thank you for this comment.*

**Specific comments for the submitted paper:**

**Introduction section:**

**1. Page 5, line 98: sentence should be written in past tense since the study was conducted in 2018.**

*The sentence has been rewritten in the past tense.*

**2. Authors should briefly describe why injectable antibiotics are the treatment of choice for serious bacterial infections.**

*Infections are a major cause of neonatal deaths. A substantial number of neonatal infections are bacterial in origin, which can be treated by injectable antibiotics, thus preventing many deaths. Recently, ANISA, a community-based study from South-East Asia, reported a cause in 26% of the PSBI episodes (16% bacterial and 12% viral) (Saha et al. Lancet 2018). It also reported that among babies with possible serious bacterial infection (PSBI) who died with an attributable cause, 92% were bacterial. Thus, injectable antibiotics remain the primary WHO recommended treatment for neonatal sepsis (Ref 8), which is mentioned in the manuscript.*

*We have modified the text in paragraphs 1 and 2 of the introduction section to highlight this and have added two references Saha et al. 2018 and WHO Global Report on sepsis 2020, in response to the reviewer’s comment.*

**3. Is there a specific reason why the North and South Ubangi provinces of DRC were chosen as study sites? A brief motivation is required.**

*We chose these provinces because they typify rural areas of the DRC. In addition, we have an existing research infrastructure there. This includes experienced research nurses, a data entry system and supervisory personnel. A statement about this motivation has been added to the text.*

**4. The importance of a mother`s immunity and neonatal vaccination in decreasing the burden of PSBI should be discussed in the introduction section.**

*The immunity of a mother may impact the likelihood of the acquisition and severity of selected neonatal diseases. However, the majority of neonatal bacterial infections are not known to be influenced by maternal immunity (the exception might be group B streptococcus). The same cannot be said for viral infections. We acknowledge that the signs and symptoms of PSBI may result from viral disease (Saha et al. Lancet 2018). However, the focus of this manuscript is the recognition and treatment of infants with these signs. Factors that might influence the prevalence of signs were not considered and would not influence the need to recognize and treat. Therefore, we did not include this information in the introduction section. Vaccination for the prevention of bacterial disease does not occur routinely during early infancy in this part of the DRC.*

**Method section:**

**Training and refresher training**

**1. Page 11, line 206. Sentence should be written in past tense.**

**….”which was organized by”.. should be revised to… “which were organized by”..**

*The sentence has been rewritten in the past tense.*

**Community surveillance and identification of sick young infants**

**2. Page 13, line 263. Sentence needs to be revised.**

*The sentence has been revised.*

**Results section:**

**1. Page 16, line 358. Sentence needs to be revised.**

*The sentence has been revised.*

**Comments from Reviewer #4**

**In the Abstract introduction, the author mentioned that injectable antibiotics may not be feasible in many low resource settings. Is there any reason, e.g access? Please indicate.**

*A sentence has been added to the Introduction section that listed reasons for non-referral. This sentence has been added to the Introduction section of the body of the manuscript only because of word limitations in the Abstract.*

**The author should add the WHO recommendation in the introduction and not just reference it.**

*Thank you for this suggestion. However, we respectfully disagree. The details of the WHO recommendations appear in the Methods. Repeating these in the Introduction section would lengthen the Introduction section, and hence the manuscript, without adding crucial detail. We have added a sentence to the Methods section highlighting that the study recommendations are consistent with the WHO guidelines.*

**Comments from Reviewer #5**

**The author could include a subheading in the methods with study definitions to describe what is meant by pneumonia, severe pneumonia, critical illness, presumed serious bacterial infection, young infant age ranges used, adequate treatment, etc. This may eliminate having to provide the definitions under each table in the article**

*Thank you for this suggestion. We have eliminated the redundancy by including the definitions in Table 1 only and removed them from footnotes under other tables.*

**The age range of a young infant is included at every mention throughout the document which need not be so if defined at the beginning of the article.**

*Thank you for this suggestion. We have removed the redundancy.*

**Abstract:
This a mixed methods longitudinal study but in the results, no mention is made of the salient findings from the qualitative side of the study**

*Thank for this observation. As noted in response to a similar question from Reviewer #1, we have provided further details in the Methods section about the qualitative analyses.*

**Introduction:
Line 99: should be “per 1000 live births”**

*“live births” has been added.*

**Data Analysis:
lines 343-347: was treatment failure (with the same/similar criteria as the secondary outcomes) a separate secondary outcome?**

*Treatment failure was a composite outcome of death, clinical deterioration, or signs signifying the inadequacy of the original treatment and/or the need to change treatment course. The sentence has been rewritten for clarity.*

**Results:
As this was a mixed method intervention study, what were the findings from the interviews/focus groups of the families?**

*We have added a sub-section summarizing information from the interviews and focus group discussions with community members in the results section under a sub-heading of the Methods, “Barriers to health care seeking”. These results were included in the Methods section because the discussions occurred during the preparatory phase of the study and informed the implementation strategy that was tested.*

**What were the reasons given by parents for refusal of transfer to a health facility?**

*We did not record reasons for the refusal of transfer. However, we assume that the reasons correspond to those given during the focus group discussion and listed in a new Table 2 that were not impacted by resources provided during the study (e.g., free medication).*

**lines 361-364 reword the sentence to simplify it**

*This sentence has been rewritten.*

**lines 381 -397: Table 2 and 3 summarise your results of the primary and secondary outcomes so there is no need to mention all of the findings in the text.**

*Thank you for this comment. As information for the PSBI sub-categories was reported in tables, we consolidated important information in the text and reduced the burden of detail in the text in the Results section.*

**The day 4 and day 8 follow up would imply that a certain number of antibiotic doses were taken. This could be cumulatively summarized for the clinical syndromes analysed.**

*Cumulative data for each syndrome appear in Table 3. A summary statement has been added to the text of the Results.*

**Provide the overall case fatality rate of those infants hospitalized**

*A sentence reporting the CFR among hospitalized infants has been added.*

**Discussion:
lines 447-448: “whereas from 23% errors in dose calculation and 15% classification errors on assessment were reported from MaMoni project, Bangladesh” remove the word “from”**

*The word has been removed.*

**Line 438: remove the reference in the middle of the statement, it has been referenced from the previous statement**

*The redundancy in the text has been removed.*

**Lines 456-457: the other research sites have been mentioned in the previous paragraph, there is no need to re-list them if referenced**

*The redundancy has been removed.*

**Comments from the Editor**

**1. Please ensure that your manuscript meets PLOS ONE's style requirements, including those for file naming. The PLOS ONE style templates can be found at**

**https://journals.plos.org/plosone/s/file?id=wjVg/PLOSOne_formatting_sample_main_body.pdf and**

[**https://journals.plos.org/plosone/s/file?id=ba62/PLOSOne_formatting_sample_title_authors_affiliations.pdf**](https://journals.plos.org/plosone/s/file?id=ba62/PLOSOne_formatting_sample_title_authors_affiliations.pdf)

*The manuscript now adheres to POLS ONE’s style requirements.*

**2. Please review your reference list to ensure that it is complete and correct. If you have cited papers that have been retracted, please include the rationale for doing so in the manuscript text, or remove these references and replace them with relevant current references. Any changes to the reference list should be mentioned in the rebuttal letter that accompanies your revised manuscript. If you need to cite a retracted article, indicate the article’s retracted status in the References list and also include a citation and full reference for the retraction notice.**

***The reference list has been reviewed with this advice in mind. All references are current and relevant.***

**3. We note that the grant information you provided in the ‘Funding Information’ and ‘Financial Disclosure’ sections do not match.**

**When you resubmit, please ensure that you provide the correct grant numbers for the awards you received for your study in the ‘Funding Information’ section.**

*On the advice of the WHO, we have revised the funding information section as follows:*

*The study was funded by the Bill and Melinda Gates Foundation through a grant to the World Health Organization. The funder had no role in study design, data collection and analysis, decision to publish, or preparation of the manuscript.*

**4. Thank you for stating the following in the Competing Interests section:**

**The authors declare no conflict of interest. Rajiv Bahl and Yasir Bin Nisar are staff members of the World Health Organization. The expressed views and opinions do not necessarily express the policies of the World Health Organization**

**Please confirm that this does not alter your adherence to all PLOS ONE policies on sharing data and materials, by including the following statement: "This does not alter our adherence to PLOS ONE policies on sharing data and materials.” (as detailed online in our guide for authors http://journals.plos.org/plosone/s/competing-interests). If there are restrictions on sharing of data and/or materials, please state these. Please note that we cannot proceed with consideration of your article until this information has been declared.**

**Please include your updated Competing Interests statement in your cover letter; we will change the online submission form on your behalf.**

*An updated Competing Interests statement has been included:*

*Rajiv Bahl and Yasir Bin Nisar are staff members of the World Health Organization. The expressed views and opinions do not necessarily express the policies of the World Health Organization. This does not alter our adherence to PLOS ONE policies on sharing data and materials.*

**5. We note that you have indicated that data from this study are available upon request. PLOS only allows data to be available upon request if there are legal or ethical restrictions on sharing data publicly. For more information on unacceptable data access restrictions, please see http://journals.plos.org/plosone/s/data-availability#loc-unacceptable-data-access-restrictions.**

**In your revised cover letter, please address the following prompts:**

**a) If there are ethical or legal restrictions on sharing a de-identified data set, please explain them in detail (e.g., data contain potentially sensitive information, data are owned by a third-party organization, etc.) and who has imposed them (e.g., an ethics committee). Please also provide contact information for a data access committee, ethics committee, or other institutional body to which data requests may be sent.**

**b) If there are no restrictions, please upload the minimal anonymized data set necessary to replicate your study findings as either Supporting Information files or to a stable, public repository and provide us with the relevant URLs, DOIs, or accession numbers. For a list of acceptable repositories, please see http://journals.plos.org/plosone/s/data-availability#loc-recommended-repositories.**

**We will update your Data Availability statement on your behalf to reflect the information you provide.**

*The following is an updated Data Availability statement:*

*All relevant data are within the manuscript.*

**6. Please upload a copy of Supporting Information Supplementary Table 1 and 2 which you refer to in your text on page 28.**

*These tables have been uploaded.*
