# Supplementary material for: Simplified antibiotic regimens for young infants with possible serious bacterial infection when the referral is not feasible in the Democratic Republic of the Congo
Source: PLoS One. 2022 Jun 30;17(6):e0268277. doi: 10.1371/journal.pone.0268277 (PMC9246187; doi:10.1371/journal.pone.0268277)
Supplement: Attachment — Submitted filename: Response to Reviewers.docx [file pone.0268277.s006.docx]

Nusrat Homaira

Academic Editor

PLOS ONE

Re: PONE-D-20-38722R1

Simplified antibiotic regimens for young infants with possible serious bacterial infection when referral is not feasible in the Democratic Republic of the Congo

Dear Homaria:

We thank you and the reviewers for your review of our manuscript. Because of the positive nature of the review, and at your request, we are submitting a revised manuscript. The following is a detailed list of the revisions and/or our responses.

We believe that our responses to the helpful advice from the reviewers and subsequent edits have improved the manuscript. We hope that you will now deem it acceptable for publication. We look forward to your further consideration.

Sincerely,

Adrien Lokangaka, on behalf of the authors

**Comments from Reviewer #1**

**In methodology, it would be great if authors will describe and elaborate the qualitative component such as: how many FGDs and KII were conducted? What was the size of each FGD? Where FGDs were conducted etc..**

*As stated in the manuscript (see line 184), we conducted 20 FGDs and 15 in-depth interviews.*

We have added the following: *In each FGD 8 to 10 people participated. The discussion took place at the health center where people were convened. The selection of participants for the FGDs was guided by the health center nurses and the information provided by the CHWs. The criteria of homogeneity of residence, age, marital status, and level of education were also taken into account.*

**Comments from Reviewer #5**

**Thank you for the revised manuscript. Some minor grammatical changes suggested:**

**1-Line 447: rather say " as an outpatient" or "on an outpatient basis"**

*Thank you for this catch. We have corrected to “on an outpatient basis”.*

**2-Line 429: insert "and" between the two categories of infants in the sentence**

*Young infants 7-59 days of age with only fast breathing represented two-fifths of all sick young infants and were treated on an outpatient basis without referral to a hospital.*

**3-Line 450: the reference number at the end of the sentence**

*Reference number moved to the end of the sentence.*

**4-Be consistent with the text format throughout the document especially Table 2**

*Thank you for this suggestion. Table 2 has been formatted accordingly.*
