# Supplementary material for: Simplified antibiotic regimens for young infants with possible serious bacterial infection when the referral is not feasible in the Democratic Republic of the Congo
Source: PLoS One. 2022 Jun 30;17(6):e0268277. doi: 10.1371/journal.pone.0268277 (PMC9246187; doi:10.1371/journal.pone.0268277)
Supplement: Attachment — Submitted filename: Reviewer comments.pdf [file pone.0268277.s003.pdf]

Reviewer comments:

The reviewed manuscript is entitled: "**Simplified antibiotic regimens for young infants with possible serious bacterial infection when referral is not feasible in the Democratic Republic of the Congo**".

The manuscript is easy to understand, very descriptive, informative and generally well-written. My comments are as follows:

Specific comments for the submitted paper:

Introduction section:

1. Page 5, line 98: sentence should be written in past tense since the study was conducted in 2018.
2. Authors should briefly describe why injectable antibiotics are the treatment of choice for serious bacterial infections.
3. Is there a specific reason why the North and South Ubangi provinces of DRC were chosen as study sites? A brief motivation is required.
4. The importance of a mother`s immunity and neonatal vaccination in decreasing the burden of PSBI should be discussed in the introduction section.

Method section:

Training and refresher training

1. Page 11, line 206. Sentence should be written in past tense.

   …."which **was** organized by".. should be revised to… "which **were** organized by"..

Community surveillance and identification of sick young infants

2. Page 13, line 263. Sentence needs to be revised.

Results section:

1. Page 16, line 358. Sentence needs to be revised.

Overall, I recommend that the manuscript be accepted for publication, provided the above-mentioned suggestions are satisfied.
