# Supplementary material for: Simplified antibiotic regimens for young infants with possible serious bacterial infection when the referral is not feasible in the Democratic Republic of the Congo
Source: PLoS One. 2022 Jun 30;17(6):e0268277. doi: 10.1371/journal.pone.0268277 (PMC9246187; doi:10.1371/journal.pone.0268277)
Supplement: Attachment — Submitted filename: Revised Reviewer comments .pdf [file pone.0268277.s005.pdf]

Reviewer comments:

The reviewed manuscript is entitled: “**Simplified antibiotic regimens for young infants with possible serious bacterial infection when referral is not feasible in the Democratic Republic of the Congo**”.

The manuscript is easy to understand, very descriptive, informative and generally well-written.

Decision on the submitted paper:

I am satisfied with the revisions made to the manuscript. I therefore recommend that the submitted paper is suitable for publication in the *PLOS ONE* journal.
